# Supplementary material for: Compensatory action of different types of cis-regulatory elements buffers the striped expression of Drosophila pair-rule genes
Source: Development. 2025 Dec 19;152(24):dev204872. doi: 10.1242/dev.204872 (PMC12752513; doi:10.1242/dev.204872)
Supplement: Supplementary information [file develop-152-204872-s1.pdf]

Fischer et al. Sup. Figure S1

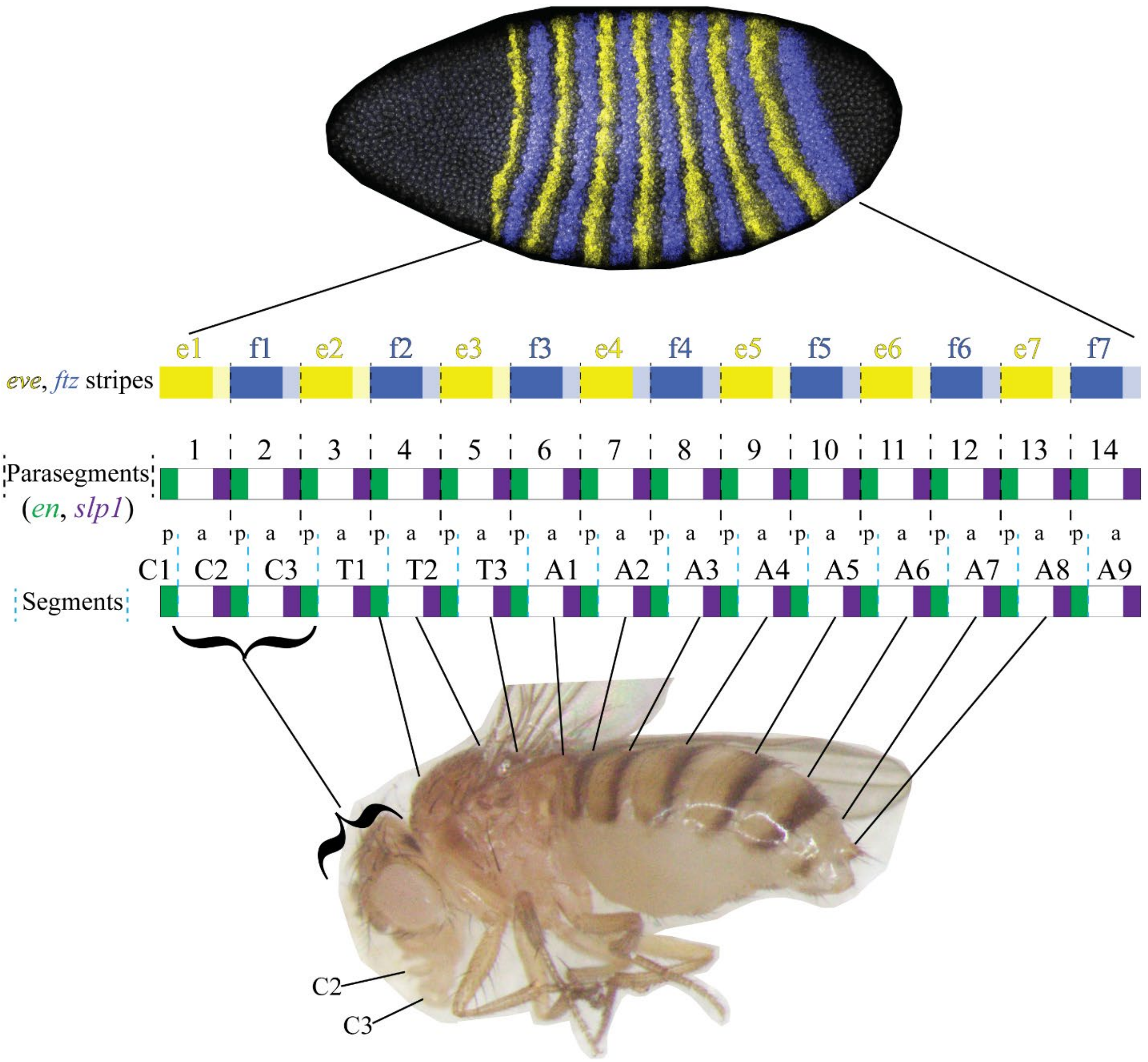

Fig. S1. Schematic of parasegments relative to *ftz* stripes, *eve* stripes, and corresponding body segments. The pair-rule genes *even-skipped* (*eve*; blue) and *fushi tarazu* (*ftz*; yellow) are expressed in alternating sets of seven stripes across the trunk of blastoderm embryo (top; *in situ* hybridization chain reaction with probes for *eve* and *ftz*; nuclei stained with Hoechst). The stripes of *eve* define the odd-numbered parasegments of the blastoderm and the stripes of *ftz* define the even-numbered parasegments of the blastoderm. The partially shaded region of the *ftz*/*eve* stripe schematic represents the shrinking of the stripe away from the posterior near the end of stage 5. The segment polarity gene *engrailed* (*en*; green) is expressed on the anterior of the parasegment and *sloppy-paired 1* (*slp1*; purple) is expressed on the posterior of the parasegment. Dotted black lines represent the boundaries of the 14 parasegments. The true segments are slightly offset from the parasegments. Dotted blue lines represent the boundaries of the segments. The anterior and posterior compartments of the true segments are labeled above the segment schematic with a and p, respectively.

Fischer et al. Sup. Figure S2

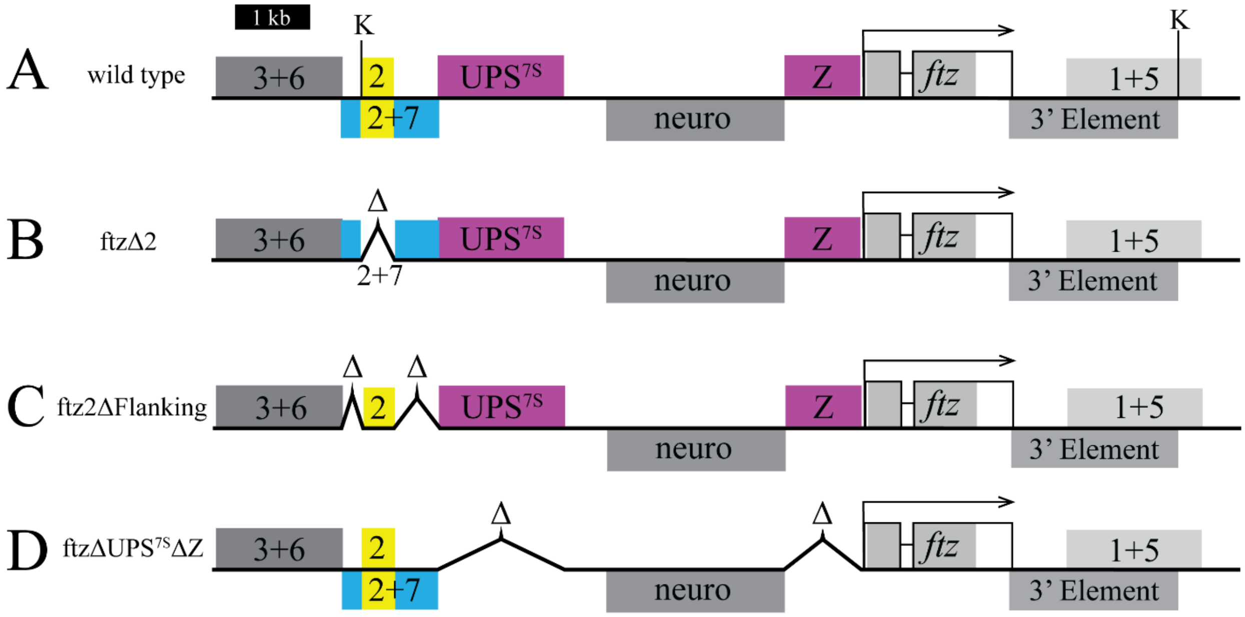

Fig. S2. Summary schematic of *ftz* locus for each of the *ftz* genome edits in this study. A) wild type *ftz* contains two seven-stripe CREs: UPS^{7S} and the zebra element (purple). Additionally, stripe-specific CREs have been previously identified for stripes 3+6 (gray), a strong stripe 2 CRE (yellow), 2+7 (blue with yellow indicating overlap with 2), 1+5 (light gray), and a 3' element (gray) that may direct expression in stripe 5. B) The *ftzΔ2* mutant has the strong stripe 2 413 bp element (yellow) precisely deleted with CRISPR-Cas9. The remainder of the 2+7 is left otherwise intact. C) The *ftz2ΔFlanking* mutant has regions of the 2+7 element (blue) flanking the strong stripe 2 element (yellow) deleted. The strong stripe 2 413 bp element remains otherwise intact. D) The *ftzΔUPS^{7S}ΔZ* double mutant has both the UPS^{7S} and Z elements (purple) deleted. All of the stripe-specific elements remain.

Development • Supplementary information

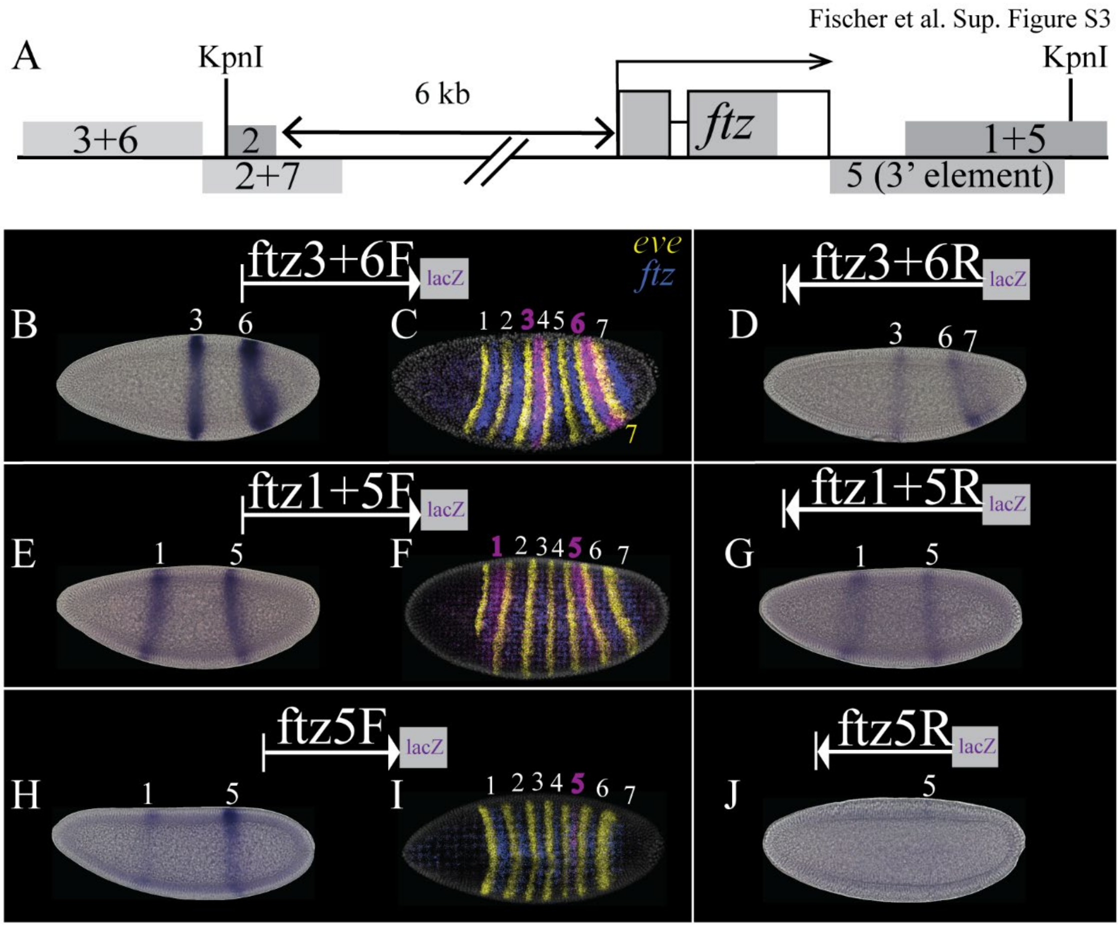

Fig. S3. Expression of *ftz* stripe-specific CREs reflects endogenous stripe expression. (A) Schematic showing stripe-specific CREs. **(B-J)** Digoxygenin-labeled probes for chromogenic *in situ* hybridization of embryos containing transgenes for ftz3+6. (B, D) ftz1+5 (E, G) and (H, J) ftz5 (3'Element) in either the forward or reverse orientation, as indicated. (C, F, I) Fluorescent confocal images of *eve*, *ftz*, and *lacZ* expression detected by *in situ* HCR in indicated transgenic reporter lines: *eve*, yellow; *ftz*, blue; *lacZ*, purple. White numbers above embryos indicate *ftz* stripes, purple numbers indicate *lacZ* co-expression. ftz3+6 was renamed from ftz_(-7) (Schroeder et al., 2011). ftz1+5 was renamed from ftz_(+3) (Schroeder et al., 2011). ftz5 was renamed from the 3' Element. See Fig. S2 and Table S1 for details on these CREs.

Fischer et al. Sup. Figure S4

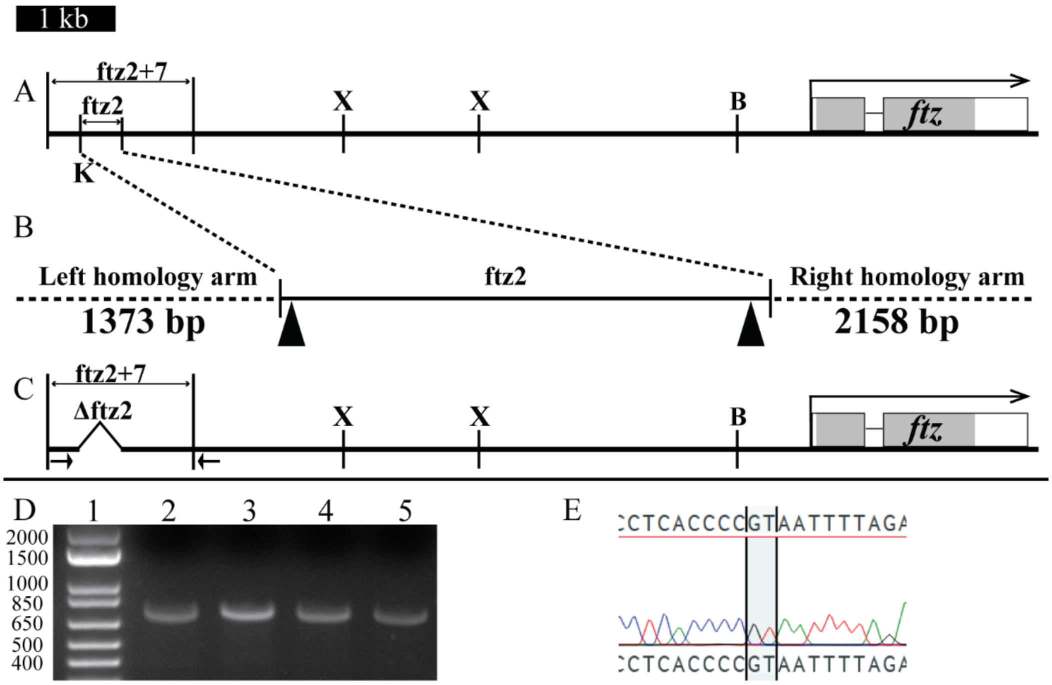

Fig. S4. Generation of the *ftzΔ2* allele by precise deletion of the ftz2 CRE using CRISPR/ Cas9 with an HDR template. (A) Schematic of the genomic region upstream relative to the *ftz* transcription unit (*ftz*, black arrow represents transcription from basal promoter, boxes represent exons with CDS in gray, UTR in white, black line represents the one intron). K: KpnI site; X: XbaI site; B: BalI site. Drawn to scale (scale bar indicates 1 kilobase). Genomic regions of ftz2 and ftz2+7 are labeled. (B) Zoom in of the ftz2 fragment with black arrowheads indicating the CRISPR/Cas9 gRNA target sites. Homology arms of the repair template are indicated on either side. (C) Schematic of the *ftzΔ2* allele resulting from the incorporation of *pUC19-ftzΔ2* following CRISPR/Cas9-induced double-stranded breaks. Arrows represent the location of the MF143 and Dm_UES_2F primer binding sites. (D) PCR screen used to identify deletion events with the primers MF143 and Dm_UES_2F to amplify a 786 bp fragment in the absence of the ftz2 region (lanes 2-5). Lane 1: Invitrogen 1 kb Plus (E) Sequencing result from a mutant identified from PCR screen. Highlight indicates the junction between the left and right homology arms of the HDR template, indicating a precise deletion.

Development • Supplementary information

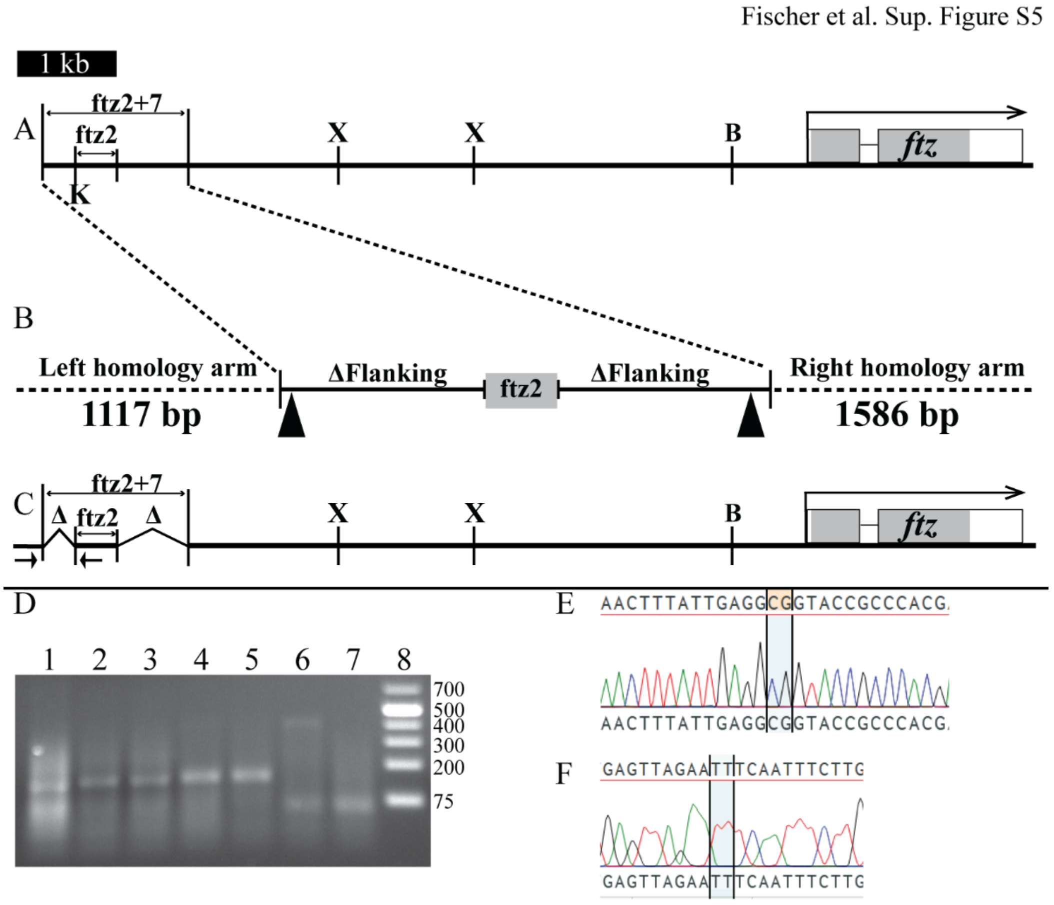

Fig. S5. Generation of the *ftz2Δ*Flanking allele by knock-in of the ftz2 CRE to replace ftz2 +7 using CRISPR/Cas9 and an HDR template. (A) Schematic of the genomic region upstream relative to the *ftz* transcription unit. Genomic fragments of ftz2 and ftz2+7 are labeled. (B) Zoom in of the ftz2+7 region indicating where the gRNAs targeted the regions that were intended for deletion (Δftz2+7), and the fragment to be re-incorporated (ftz2) following HDR of the CRISPR/ Cas9-induced double-stranded breaks. Homology arms of the repair template with base length are indicated on either side. (C) Schematic of the *ftz2ΔFlanking* allele resulting from incorporation of *pUC19- ftz2Δ2+7* following CRISPR/Cas9-induced double-stranded breaks. Arrows represent the location of the MF21 and MF149 primer binding sites. (D) PCR screen used to identify HDR events with the primers MF21 and MF149, which amplify a 149 bp from positive events (lanes 1-5), a 405 bp fragment from negative genomic control events (lanes 6), and a no-template negative control (lane 7). Small bands <75 bp are from primers. Lane 8 contains GeneRuler 1kb plus DNA ladder. Sequencing result from a mutant identified from PCR screen. Highlight indicates the junction between the left homology arm and ftz2 (E) and between the right homology arm and ftz2 (F).

Development • Supplementary information

Fischer et al. Sup. Figure S6

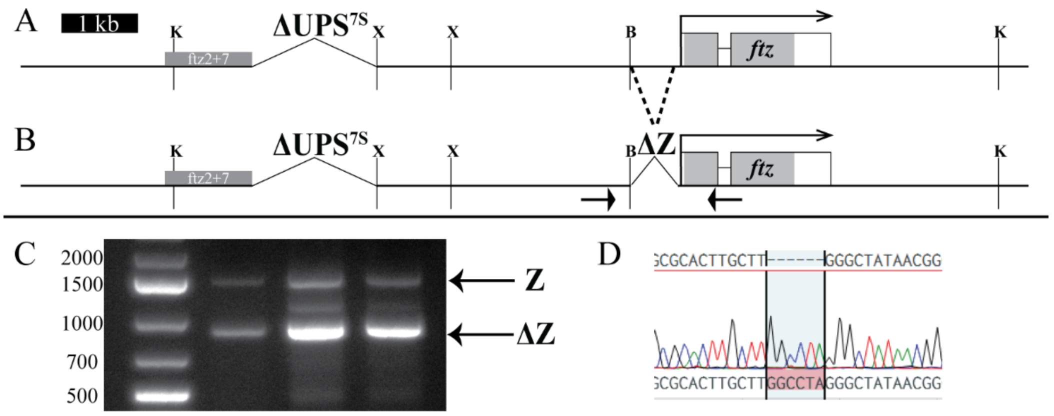

Fig. S6. Generation of the homozygous lethal *ftzΔUPS^{7S}ΔZ* allele by precisely deleting the UPS and Z CREs of *ftz*. (A-B) Schematic for deleting the *ftz* zebra CRE from the *ftzΔUPS^{7S}* background (A) to generate the double deletion mutant *ftzΔUPS^{7S}ΔZ* (B). Guide RNAs and repair template for HDR were the same as described in (Graham et al., 2021). Progeny heterozygous for *ftzΔUPS^{7S}ΔZ/TM3, Sb* were screened with PCR using the primers zebra1 and zebra 2 (B, arrows), which amplifies a 1485 bp fragment in the presence of the zebra element (C; Z arrow) or 888 bp fragment from precise deletion mutants (C; ΔZ arrow); both bands confirm heterozygosity. Positive lines were sent for sequencing to confirm the replacement of the zebra element with an AvrII restriction site (D; red highlight indicates AvrII site from HDR template).

Development • Supplementary information

Fischer et al. Sup. Figure S7

A Fluorescent intensity of ftz stripes in central 10% of w[1118], ftzΔ2, and ftz2ΔFlanking embryos

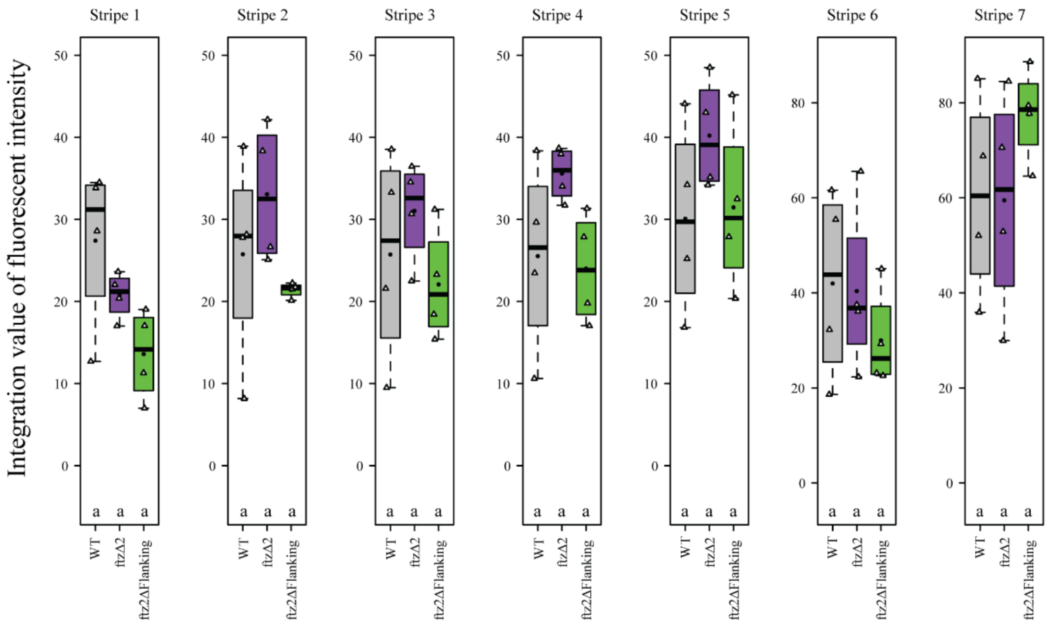

B Width of ftz stripes in central 10% of w[1118], ftzΔ2, and ftz2ΔFlanking embryos

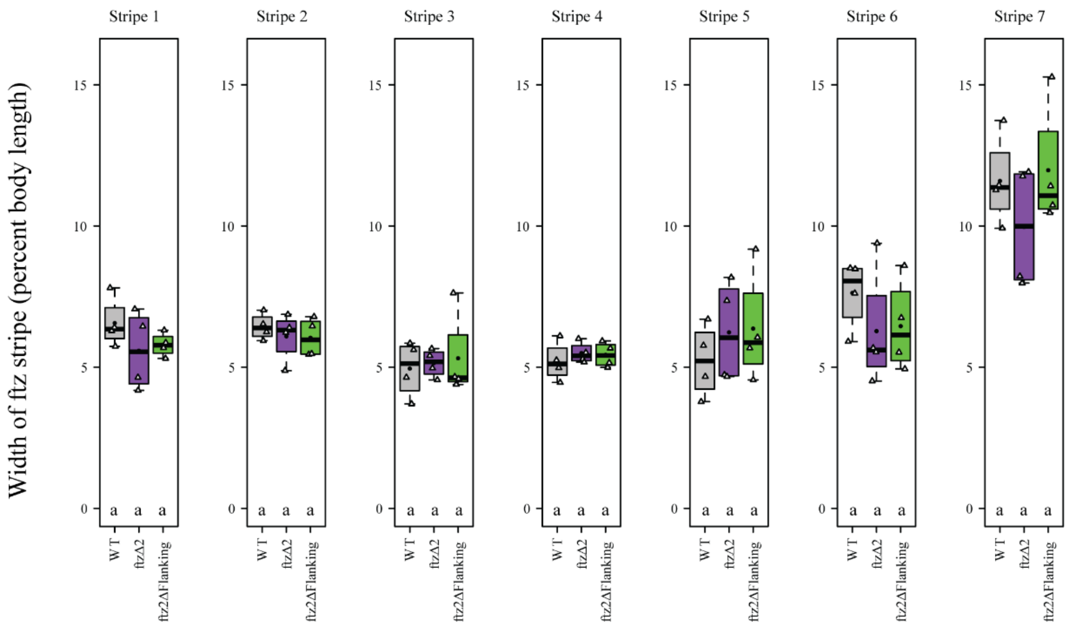

Development • Supplementary information

Fig. S7. Quantification of *ftz* stripe intensity and width from fluorescent *in situ* HCR. There is no statistically significant difference in the quantity of *ftz* in each stripe nor the size of each stripe when comparing wild type (gray), *ftzΔ2* (purple), and *ftz2ΔFlanking* (green) embryos at the end of stage 5. Sub-stage of embryo determined by degree of membrane deposition around nuclei. Data quantified from fluorescent in situ HCR experiments. Letters above X-axis label represent statistical groups, with same letter signifying no statistically significant difference. Statistical significance with multiple comparison tested with Kruskal-Wallis and post hoc tested using the criterium Fisher's least significant difference. P-value adjusted with the Bonferroni-Holm correction for multiple comparisons. Whiskers of box plot extend to the most extreme data point which is no more than range times the interquartile range from the box.

Fischer et al. Sup. Figure S8

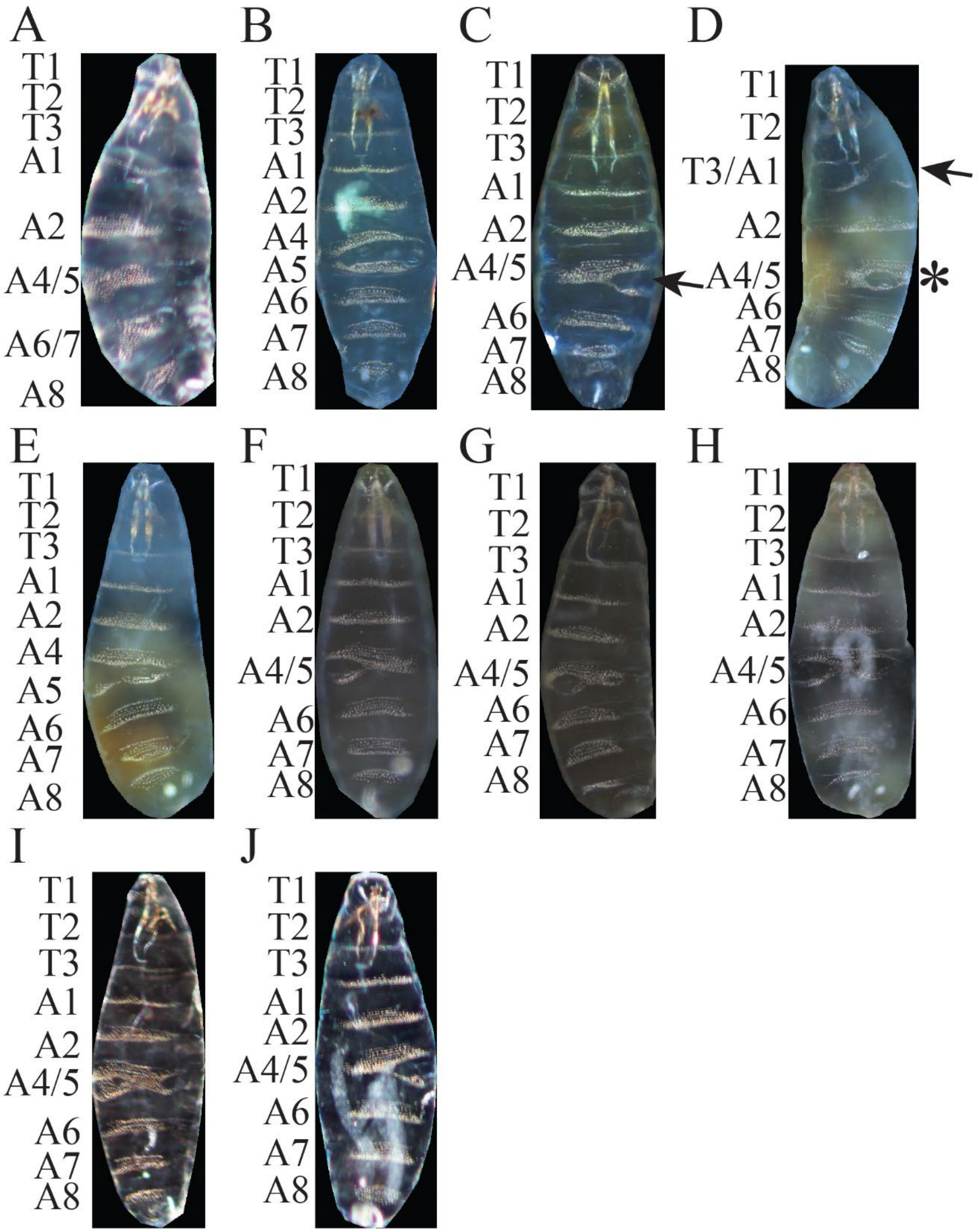

Fig. S8. Additional cuticle preparations from *ftzΔUPS^{7S}ΔZ*. Most *ftzΔUPS^{7S}ΔZ* larvae were missing abdominal segment A3 and A4 /A5 were fused (asterisk) or partially fused/ deleted (arrow).

Development • Supplementary information

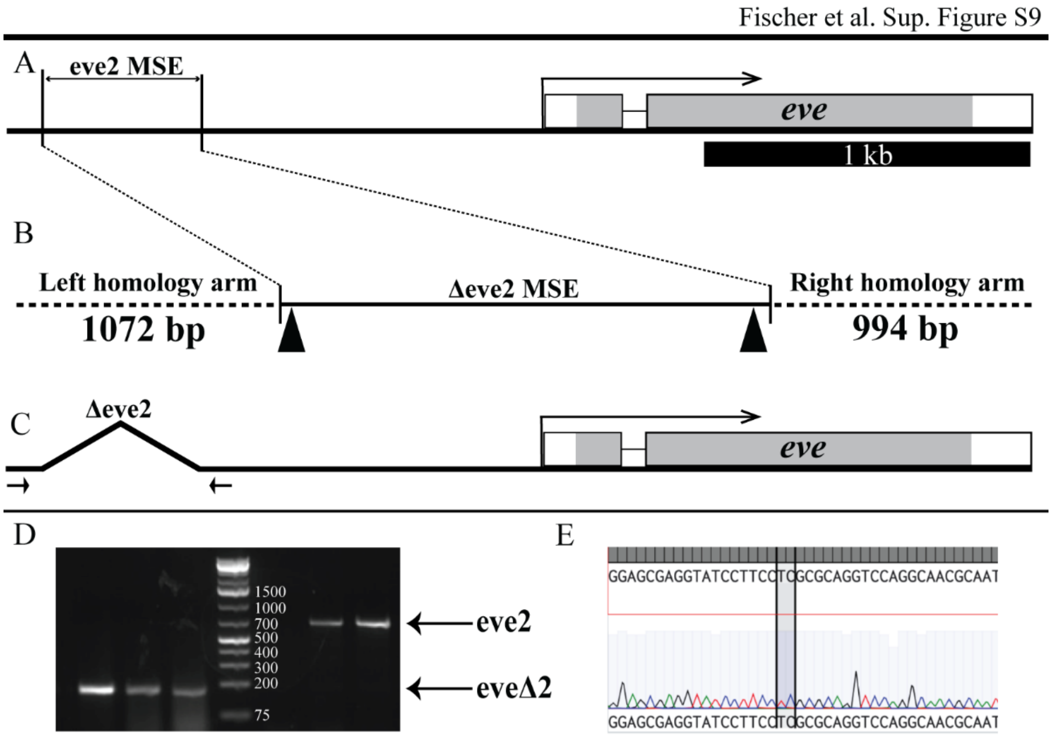

Fig. S9. Generation of the *eveΔ2* allele by precise deletion of the eve2 CRE using CRISPR/ Cas9 with an HDR template. (A) Schematic of the genomic region upstream relative to the *eve* transcription unit drawn to scale. Genomic fragment of eve2 minimal stripe enhancer (eve2 MSE) is labeled. (B) Zoom-in of the eve2 MSE region indicating where the gRNAs targeted, the 480 bp region that was intended for deletion (Δeve2 MSE), and the fragment to be re-incorporated (homology arms) following HDR of the CRISPR/Cas9-induced double-stranded breaks. Homology arms of the repair template with base length are indicated on either side. (C) Schematic of the *eveΔ2* allele resulting from incorporation of *pUC19- eveΔ2* following CRISPR/ Cas9-induced double-stranded breaks. Arrows represent the location of the MF164 and MF165 primer binding sites. (D) PCR screen used to identify HDR events with the primers MF164 and MF165, which amplify a 187 bp from positive events (lanes 1-3) and a 671 bp fragment from negative events (lanes 6-7). Lane 4 contains GeneRuler 1kb plus DNA ladder. (E) Sequencing result from a mutant identified from PCR screen. Highlight indicates the junction between the left homology arm and the right homology arm.

Development • Supplementary information

Fischer et al. Sup. Figure S10

A Fluorescent intensity of *eve* stripes in central 10% of w^{1118} versus $eve\Delta 2^{-/-}$ embryos

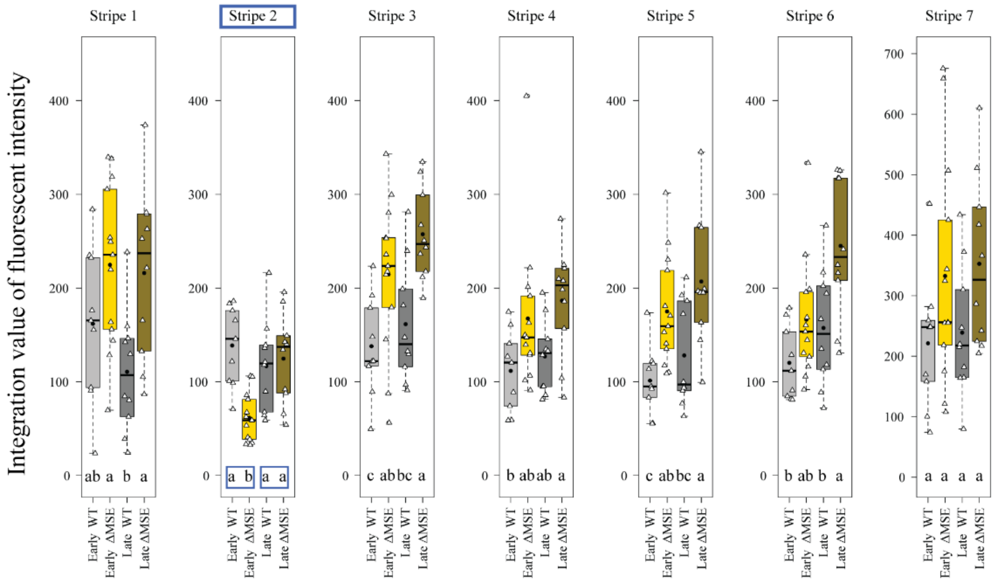

B Width of *eve* stripes in central 10% of w^{1118} versus $eve\Delta 2^{-/-}$ embryos

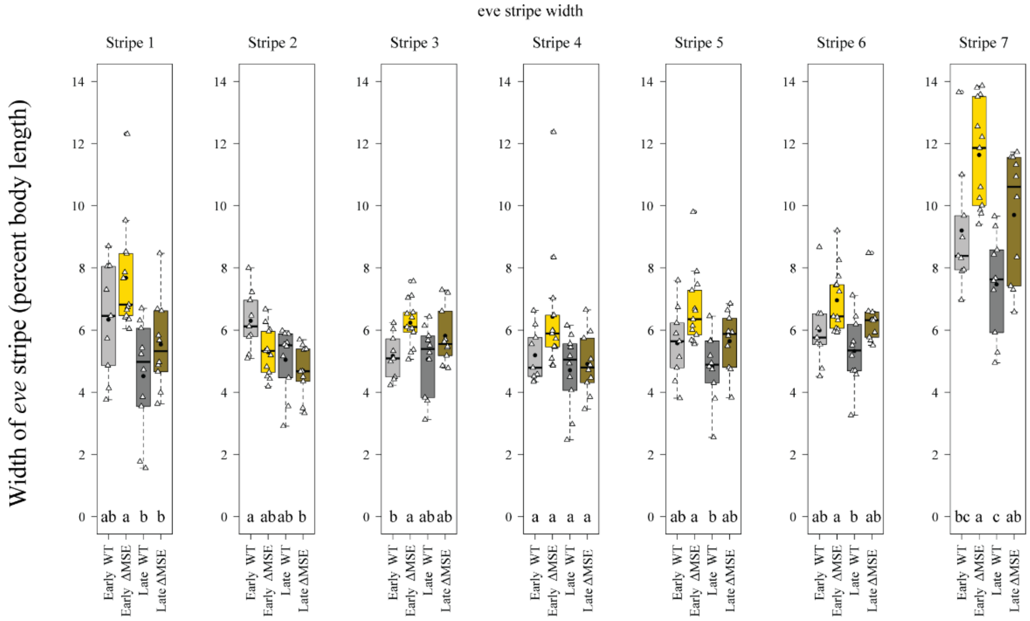

Fig. S10. Quantification of *eve* stripe intensity and width from fluorescent *in situ* HCR. Stripe 2 of *eve* is significantly lower in early stage 5 *eveΔ2* embryos (yellow) compared to wild type (gray) and reaches wild type levels by late stage 5. Hue of box plot indicates early vs late stage 5; light gray and yellow are early stage 5, and dark gray and dark yellow are late stage 5. No other stripes are significantly lower in *eveΔ2* embryos, but *eve* is higher in the mutant embryos in late stage 5 for stripes 1, 3, 5, and 6. Data quantified from fluorescent in situ HCR experiments. The width of the *eve* stripes in the mutant embryos is statistical insignificant compared to wild type for most stripes. Stripe 3 is wider in early stage 5 embryos in mutants compared to wild type, though the difference is statistically insignificant by late stage 5. Stripe 7 is wider in mutant embryos compared to wild type in both early and late stage 5 embryos. Sub-stage of embryo determined by degree of membrane deposition around nuclei. Letters above X-axis label represent statistical groups, with different letters signifying a statistically significant difference. Statistical significance with multiple comparison tested with Kruskal-Wallis and post hoc tested using the criterium Fisher's least significant difference. P-value adjusted with the Bonferroni-Holm correction for multiple comparisons. Whiskers of box plot extend to the most extreme data point which is no more than range times the interquartile range from the box.

Fischer et al. Sup. S11

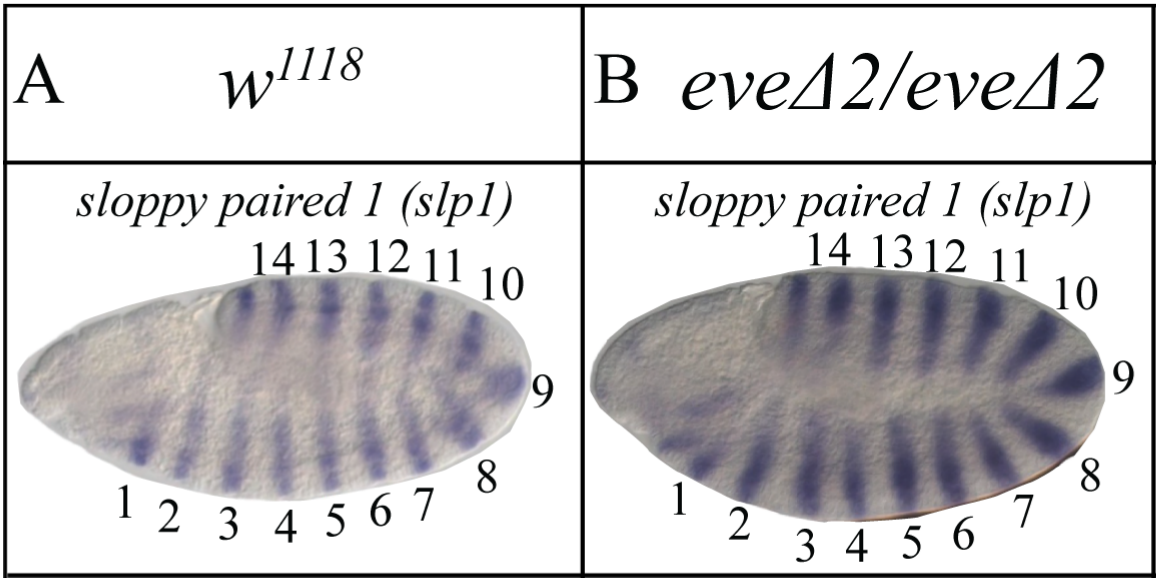

Fig. S11. Expression of *slp1* was not altered in*eveΔ2/eveΔ2* homozygotes. (A) Wildtype *slp1* expression. (B) Expression of slp1 in e*veΔ2/eveΔ2* homozygotes.

Development • Supplementary information

Fischer et al. Sup. Figure S12

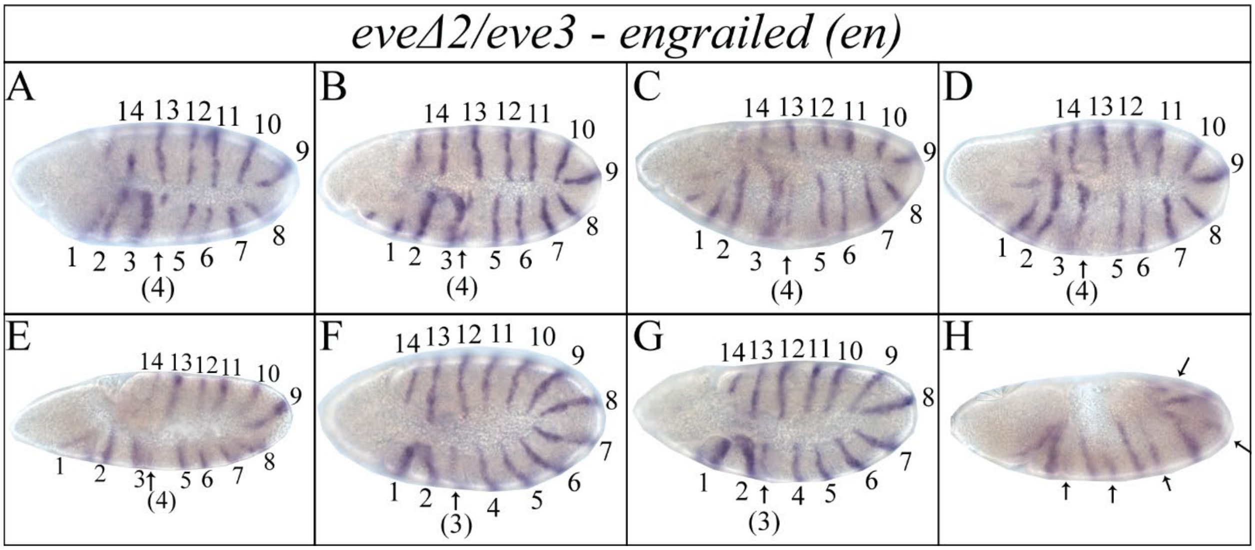

Fig. S12. Expression of *en* in *eveΔ2/eve³* transheterozygotes. Three examples were shown in Figure 6 (J-L). Here we show additional examples of embryos, to point out the variation in *en* expression in transheterozygotes.

Development • Supplementary information

Table S1. *Cis* -regulatory elements used in this study.

Name in this study	Name from previous study	Reporter or CRISPR/Cas9 deletion	Directs expression in:	Size (bp)	Description	Source
ftz2+7	ftz_(-6)	Reporter	ftz stripes 2 and 7	1240	*lacZ* reporter transgene inserted in BL 9740	Schroeder et al., 2011
ftz2	413	Reporter	ftz stripe 2	413	*lacZ* reporter transgene inserted in BL 9740	Pick et al., 1990
ftz3+6	ftz_(-7)	Reporter	ftz stripes 3 and 6	1554	*lacZ* reporter transgene inserted in BL 9740	Schroeder et al., 2011
ftz5	3' Element	Reporter	ftz stripe 5	2133	*lacZ* reporter transgene inserted in BL 9740	Hiromi 1985
ftz1+5	ftz_(+3)	Reporter	ftz stripes 1 and 5	1763	*lacZ* reporter transgene inserted in BL 9740	Schroeder et al., 2011
ΔUPS 7S	UPS	CRISPR/Cas9 deletion	ftz stripes: all seven	Δ1590	Deletion of UPS enhancer in endogenous ftz locus	Original: Hiromi 1985; UPS 7S Endogenous deletion in Fischer et al., 2024
Δzebra	zebra	CRISPR/Cas9 deletion	ftz stripes: all seven	Δ597	Deletion of zebra enhancer in endogenous ftz locus	Original: Hiromi 1985; Endogenous deletion in Graham et al., 2021
ftzΔ2	413	CRISPR/Cas9 deletion	ftz stripe 2	Δ413	Deletion of ftz2 enhancer in endogenous ftz locus	Deletion is novel to this work.
ftz2ΔFlanking	N/A	CRISPR/Cas9 deletion	ftz stripe 2 and 7	Δ827	Deletion of sequences flanking ftz2 that correspond to ftz2+7 in endogenous ftz locus (256 bp to left of ftz2, 571 bp to right of ftz2)	Deletion is novel to this work.
eveΔ2	eve minimal stripe 2 element	CRISPR/Cas9 deletion	eve stripe 2	Δ484	Deletion of eve MSE in endogenous eve locus	Original MSE reporter: Small et al., 1992 ; MSE deleted in transgene: Ludwig et al. 2005, 2011; Endogenous deletion is novel to this work.

Development • Supplementary information

Table S2. HCR quantitation of *ftz* stripe integrations and width from central 10% of wildtype, ftzΔ2, and ftz2ΔFlanking embryos.

	WT stripe integration average ± standard deviation	ftzΔ2 stripe integration average ± standard deviation	Percent (ftzΔ2 /WT)	ftz2ΔFlanking stripe integration average ± standard deviation	Percent (ftz2ΔFlanking /WT)	Kruskal-Wallis P-value
Stripe 1	27.41 ±10.16	20.77 ± 2.826	75.79 ± 29.92	13.6 ± 5.489	49.61 ± 43.3	0.09212
Stripe 2	25.76 ±12.81	33.06 ± 8.472	128.3 ± 71.8	21.41 ± 0.9026	83.09 ± 20.09	0.1054
Stripe 3	25.72 ±12.93	31.04 ± 6.185	120.7 ± 64.74	22.09 ± 6.896	85.86 ± 38.98	0.3897
Stripe 4	25.53 ±11.66	35.6 ± 3.294	139.4 ± 64.98	24.01 ± 6.705	94.05 ± 54.39	0.07748
Stripe 5	30.09 ±11.73	40.22 ± 6.802	133.7 ± 56.8	31.47 ± 10.41	104.6 ± 53.29	0.3094
Stripe 6	41.98 ±20.05	40.37 ± 18.14	96.17 ± 63.06	30.01 ± 10.49	71.48 ± 31.99	0.6939
Stripe 7	60.45 ±21.21	59.48 ± 23.57	98.39 ± 52.08	77.59 ± 9.906	128.4 ± 45.62	0.3973
	WT stripe width average ± standard deviation	ftzΔ2 stripe width average ± standard deviation	Percent (ftzΔ2 /WT)	ftz2ΔFlanking stripe width average ± standard deviation	Percent (ftz2ΔFlanking /WT)	Kruskal-Wallis P-value
Stripe 1	6.562 ± 0.8813	5.582 ± 1.389	85.07 ± 24.05	5.795 ± 0.4152	88.3 ± 24.26	0.4724
Stripe 2	6.435 ± 0.4584	6.095 ± 0.854	94.72 ± 18.56	6.045 ± 0.6833	93.94 ± 14.86	0.6939
Stripe 3	4.952 ± 0.9861	5.148 ± 0.4899	103.9 ± 13.8	5.318 ± 1.546	107.4 ± 23.56	0.926
Stripe 4	5.2 ± 0.6896	5.5 ± 0.3683	105.8 ± 21.27	5.438 ± 0.4374	104.6 ± 15.57	0.6183
Stripe 5	5.23 ± 1.274	6.235 ± 1.804	119.2 ± 37.91	6.368 ± 1.982	121.7 ± 45.49	0.6939
Stripe 6	7.63 ± 1.219	6.28 ± 2.138	82.31 ± 31.21	6.455 ± 1.616	84.6 ± 31.11	0.4909
Stripe 7	11.6 ± 1.584	9.972 ± 2.165	85.99 ± 22.05	11.98 ± 2.238	103.3 ± 23.39	0.7939

Table S3. HCR quantitation of stripe integrations and width from central 10% of wildtype and eveΔ2 embryos.

	early WT stripe integration average ± standard deviation	late WT stripe integration average ± standard deviation	Percent (early WT/late WT)	early eveΔ2 stripe integration average ± standard deviation	late eveΔ2 stripe integration average ± standard deviation	Percent (early eveΔ2/late eveΔ2)	Percent (early eveΔ2/early WT)	Percent (late eveΔ2/late WT)	Kruskal-Wallis P-value
Stripe 1	161.9 ± 82.44	110.7 ± 64.76	146.2 ± 113.4	224.8 ± 87.21	216.1 ± 91.45	104 ± 59.72	138.9 ± 88.89	195.2 ± 140.9	0.01488
Stripe 2	138.7 ± 42.35	116.8 ± 49.02	118.8 ± 61.63	61.62 ± 26.28	124.7 ± 48.16	49.4 ± 28.43	44.41 ± 23.3	106.8 ± 60.89	0.0006972
Stripe 3	137.9 ± 53.82	161.5 ± 62.84	85.41 ± 47.06	214.6 ± 81.06	257.5 ± 48.55	83.34 ± 35.18	155.6 ± 84.52	159.4 ± 68.94	0.002476
Stripe 4	111.7 ± 43.34	127.1 ± 38.55	87.89 ± 43.23	167.5 ± 81.32	186.6 ± 57.52	89.75 ± 51.62	150 ± 93.2	146.9 ± 63.49	0.014
Stripe 5	101.3 ± 36.68	128.1 ± 55.56	79.04 ± 44.68	175 ± 59.04	207 ± 69.81	84.52 ± 40.33	172.8 ± 85.5	161.6 ± 88.78	0.001749
Stripe 6	120.2 ± 39.45	157.4 ± 62.3	76.37 ± 39.27	166.3 ± 64.46	245 ± 74.19	67.89 ± 33.39	138.3 ± 70.27	155.6 ± 77.57	0.003772
Stripe 7	221.4 ± 113.3	239 ± 106.2	92.62 ± 62.78	332.6 ± 185.8	352.8 ± 140.6	94.29 ± 64.69	150.3 ± 113.8	147.6 ± 88.11	0.1492

	early WT stripe width average ± standard deviation	late WT stripe width average ± standard deviation	Percent (early WT/late WT)	early eveΔ2 stripe integration width ± standard deviation	late eveΔ2 stripe width average ± standard deviation	Percent (early eveΔ2/late eveΔ2)	Percent (early eveΔ2/early WT)	Percent (late eveΔ2/late WT)	Kruskal-Wallis P-value
Stripe 1	6.34 ± 1.821	4.519 ± 1.808	140.3 ± 69.1	7.679 ± 1.742	5.54 ± 1.443	138.6 ± 47.88	121.1 ± 44.33	122.6 ± 58.53	0.001726
Stripe 2	6.3 ± 0.959	5.046 ± 1.063	124.9 ± 32.45	5.289 ± 0.7667	4.699 ± 0.8221	112.6 ± 25.57	83.96 ± 17.65	93.12 ± 25.5	0.01496
Stripe 3	5.17 ± 0.7193	5.058 ± 1.119	102.2 ± 26.71	6.235 ± 0.7445	5.815 ± 0.9091	107.2 ± 21.09	120.6 ± 22.11	115 ± 31.14	0.02571
Stripe 4	5.191 ± 0.8574	4.71 ± 1.221	110.2 ± 33.88	6.429 ± 2.02	4.911 ± 0.981	130.9 ± 48.74	123.9 ± 43.96	104.3 ± 34.12	0.03728
Stripe 5	5.577 ± 1.194	4.811 ± 1.089	115.9 ± 36.12	6.69 ± 1.193	5.644 ± 0.9454	118.5 ± 29	120 ± 33.42	117.3 ± 33.04	0.007623
Stripe 6	5.993 ± 1.219	5.329 ± 1.105	112.5 ± 32.67	6.959 ± 1.023	6.357 ± 0.8394	109.5 ± 21.63	116.1 ± 29.14	119.3 ± 29.33	0.009891
Stripe 7	9.203 ± 2.031	7.471 ± 1.636	123.2 ± 38.3	11.63 ± 1.719	9.704 ± 2.056	119.9 ± 30.96	126.4 ± 33.57	129.9 ± 39.58	0.0004391

Development • Supplementary information

Table S4. Viability of *ftz* and *eve* stripe 2 alleles.

25°C	Strain	# eggs scored	% hatched (#)	% pupae (#)	% adult (#)	**final % adult survival**
Rep 1	w^{1118}	100	93 (93)	75 (70)	90 (63)	63
Rep 2	w^{1118}	100	85 (85)	74 (63)	93 (59)	59
Rep 3	w^{1118}	100	92 (92)	85 (78)	92 (72)	72
Rep 4	w^{1118}	100	77 (77)	78 (60)	93 (56)	56
Average						**62.5**
Rep 1	*eveΔ2*	100	65 (65)	42 (27)	70 (19)	19
Rep 2	*eveΔ2*	100	62 (62)	35 (21)	63 (14)	14
Rep 3	*eveΔ2*	100	54 (54)	65 (34)	68 (23)	23
Rep 4	*eveΔ2*	100	56 (56)	70 (39)	62 (24)	24
Average						**20**
Rep 1	*ftzΔ2*	100	85 (85)	74 (63)	78 (49)	49
Rep 2	*ftzΔ2*	100	83 (83)	77 (64)	75 (47)	47
Rep 3	*ftzΔ2*	100	76 (76)	78 (59)	69 (41)	41
Average						**45.7**

**28°C**	Strain	# eggs scored	% hatched (#)	% pupae (#)	% adult (#)	**final % adult survival**
Rep 1	w1118	100	97 (97)	40 (38)	95 (38)	38
Rep 2	*w1118*	100	78 (78)	72 (56)	90 (50)	50
Average						**44**
Rep 1	*eveΔ2*	100	68 (68)	44 (30)	63 (19)	19
Rep 2	*eveΔ2*	100	72 (72)	29 (22)	55 (12)	12
Average						**15.5**
Rep 1	*ftzΔ2*	100	65 (65)	43 (28)	36 (10)	10
Rep 2	*ftzΔ2*	100	54 (54)	50 (27)	37 (10)	10
Average						**10**

Development • Supplementary information

Table S5. Transheterozygous survival for *ftz* alleles

`	Cross #	Male Sb	Male Non Sb	Female Sb	Female Non-Sb	Total Sb	Total Non-Sb
ftzΔ2/ftzΔ2 x ftz5/TM3,Sb	1	**21**	31	37	55	58	86
	2	4	2	6	15	10	17
	3	49	53	35	38	84	91
	4	13	17	14	20	27	37
	Total	87	103	92	128	**179 Sb**	**231 non-Sb**
ftz5/TM3,Sb x ftzΔ2/ftzΔ2	1	26	41	40	30	66	71
	2	9	14	14	14	23	28
	Total	35	55	54	44	**89 Sb**	**99 non-Sb**
ftzΔ2/ftzΔ2 x ftz11/TM3,Sb	1	31	0	66	0	97	0
	2	1	0	7	0	8	0
	3	99	0	111	0	210	0
	4	40	0	46	0	86	0
	Total	171	0	230	0	**401 Sb**	**0 non-Sb**
ftz11/TM3,Sb x ftzΔ2/ftzΔ2	1	38	0	37	0	75	0
	2	41	0	29	0	70	0
	Total					**145 Sb**	**0 non-Sb**
ftz5/TM3, Sb x ftz11/TM3,Sb	Total	32	0	50	0	**82 Sb**	**0 non-Sb**
ftz11/TM3,Sb x ftz5/TM3,Sb	Total	27	0	39	0	66 Sb	**0 non-Sb**

Development • Supplementary information

Table S6. Transheterozygous survival for *eve* alleles

Parents (F x M)	Cross #	Male Cy	Male Non Cy	Female Cy	Female Non-Cy	Total Cy	Total Non-Cy
eveΔ2/eveΔ2 x eve1/CyO	1	**131**	17	144	35	275	52
	2	84	10	107	16	191	26
	3	68	15	97	25	165	40
	4	71	3	96	13	167	16
	5	29	7	19	7	48	14
	Total	383	52	463	96	**846 Cy**	**148 non-Cy**
eve1/CyO x eveΔ2/eveΔ2	1	155	2	172	0	327	2
	2	88	0	87	0	175	0
	3	73	0	75	2	148	2
	4	79	0	70	1	149	1
	5	31	0	40	2	71	2
	Total	426	2	444	5	**870 Cy**	**7 non-Cy**
eveΔ2/eveΔ2 x eve3/CyO	1	149	0	179	0	328	0
	2	138	0	193	0	331	0
	3	116	0	125	0	241	0
	4	87	0	98	0	185	0
	Total	490	0	595	0	**1085 Cy**	**0 non-Cy**
eve3/CyO x eveΔ2/eveΔ2	1	125	0	130	0	255	0
	2	112	0	139	0	251	0
	3	133	0	140	0	273	0
	4	88	0	99	0	187	0
	5	123	0	139	0	262	0
	Total	581	0	647	0	**1228 Cy**	**0 non-Cy**
eveΔ2/eveΔ2 x Df(eve)/CyO	1	150	0	176	0	326	0
	2	70	0	98	0	168	0
	Total	220	0	274	0	**494 Cy**	**0 non-Cy**
eve3/CyO x eve1/CyO	1	70	0	135	0	205	0
	2	84	0	92	0	176	0
	3	157	0	181	0	338	0
	Total	311	0	408	0	**719 Cy**	**0 non-Cy**

Table S7. Primers used.

Primer	Sequence	Purpose	Source
CFDN-Seq	GGGCTTTGAGTGTGTGTAGAC	Colony PCR and sequencing	Graham et al 2021
MF1	GCTCTAGAGGTACCGCCCACGAGCC	ftz2 cloning	This work
MF2	GCTCTAGAATTCTAACTCAATTTAAGTCATAAAAATAACCAAAGCATTG	ftz2 cloning and colony PCR	This work
MF3	GCTCTAGATGCCGGTGGTCGTAGGAG	ftz-6 cloning	This work
MF4	GCTCTAGATGTAATGGCAGTAATGTTTCGC	ftz-6 cloning	This work
MF21	TATCGCTTTACGACCGGAGG	ftz2ΔFlanking CRISPR screen and colony PCR	This work
MF22	GGTTCGGATCGCAGGCAATC	Sequencing	This work
MF24	GGACCCATGACACGGTGC	Sequencing	This work
MF25	ACATTCGCAGTTCAACATCTTAATC	Δ2 HDR template, sequencing, and colony PCR	This work
MF40	CAGTGAATTCGAGCTCGGTACGTGTACCCATTGACAATTTTACAAAG	ΔUPS, Δ2, and ftz2ΔFlanking HDR templates	This work
MF41	CGGCTCGAGGGTACCTCTAGAATGTTTCTCGTTCGAGGTTG	ftz_(-7) cloning	This work
MF42	TCTATTTATACTCCGGCGCTCTAGACCGGCAGCCTCAATAAAG	ftz_(-7) cloning	This work
MF43	TCTATTTATACTCCGGCGCTCTAGAATGTTTCTCGTTCGAGGTTG	ftz_(-7) cloning	This work
MF44	CGGCTCGAGGGTACCTCTAGACCGGCAGCCTCAATAAAG	ftz_(-7) cloning	This work
MF45	CGGCTCGAGGGTACCTCTAGACTATTTATTTAAATATACGAGTAAAGTAAATCGATCGAA	ftz3' element cloning	This work
MF46	TCTATTTATACTCCGGCGCTCTAGACTGCCGGCTAAGTGGACAC	ftz3' element cloning	This work
MF47	TCTATTTATACTCCGGCGCTCTAGACTATTTATTTAAATATACGAGTAAAGTAAATCGATCGAA	ftz3' element cloning	This work
MF48	CGGCTCGAGGGTACCTCTAGACTGCCGGCTAAGTGGACAC	ftz3' element cloning	This work
MF63	CGGCTCGAGGGTACCTCTAGACCGCTGCACGTTCCTCTATT	ftz_(+3) cloning	This work
MF64	TCTATTTATACTCCGGCGCTCTAGAGCCTGTCGTGTTCCTTCC	ftz_(+3) cloning	This work
MF65	TCTATTTATACTCCGGCGCTCTAGACCGCTGCACGTTCCTCTATT	ftz_(+3) cloning	This work
MF66	CGGCTCGAGGGTACCTCTAGAGCCTGTCGTGTTCCTTCC	ftz_(+3) cloning	This work
MF94	GGGCGGTACCGCCTCAATAAAGTTTTATTAGGACC	ftz2ΔFlanking HDR template	This work
MF95	GAGGCGGTACCGCCCACGAGCC	ftz2ΔFlanking HDR template	This work
MF96	CACAAGAAATTGAAATTCTAACTCAATTTAAGTCATAAAAATAACCAAAGCATTG	ftz2ΔFlanking HDR template and colony PCR	This work
MF97	TTATGACTTAAATTGAGTTAGAATTTCAATTTCTTGTGAGACGGGG	ftz2ΔFlanking HDR template and sequencing	This work
MF110	TGACGATCGACAATTGTGGCGCACCAGCCGGGAATCGA	ebony sgRNA PCR	Kane et al 2017
MF112	TTATTTTAACTTGCTATTTCTAGCTCTAAAACTGACGATCGACAATTGTGGC	ebony sgRNA PCR	Kane et al 2017
MF113	GATCGGATCGAGTCGTTAAGTC	Sequencing	This work
MF125	ACCTAGTAGGATTACGAACAAG	Sequencing	This work
MF127	AAAGTGCCCACAGATGCC	Sequencing	This work
MF138	TCGATTCCCGGCCGATGCACTGTCTTCCCGGCTCGTGGGGTTTTAGAGCTAGAAATAGCA	ftz2 sgRNA PCR	This work
MF139	AAGCATTGTCTAATGCTATTGCACCAGCCGGGAATCGA	ftz2 sgRNA PCR	This work
MF140	AATAGCATTAGACAATGCTTGTTTTAGAGCTAGAAATAGCA	ftz2 sgRNA PCR	This work
MF141	GTTAAATAAAATATTTTTCAATTAAAATCTAAAATTACGGGGTGAGGGCAGAAAC	ftzΔ2 HDR template	This work
MF142	GTAATTTTAGATTTTAATTGAAAAATATTTTATTTAAC	ftzΔ2 HDR template and sequencing	This work
Dm_UES_2F	TAGGCCTCCTTTGGTAGCCTC	ftzΔ2 HDR template and sequencing	This work
MF143	CTGTAATGGCAGTAATGTTTC	ftzΔ2 HDR template and sequencing	This work
MF144	TCTAGAGGATCCCCGGGTACTCTAGATAAATGAAGTAATCTTCAGTTTC	ftz2ΔFlanking HDR template	This work
MF145	TCGATTCCCGGCCGATGCAAGCAAAACTCCTACGACCACGTTTTAGAGCTAGAAATAGCA	ftz_(-6) sgRNA PCR	This work
MF146	TTTGCGAAACATTACTGCCAGCACCAGCCGGGAATCGA	ftz-6 sgRNA PCR	This work
MF147	TGGCAGTAATGTTTCGCAAAGTTTTAGAGCTAGAAATAGCA	ftz-6 sgRNA PCR	This work
MF149	GCCGGAATTGTTCCTAATCCCC	ftz2ΔFlanking CRISPR screen	This work
zebra1	ATTTTGGAAGTGCGTTTGTTG	ΔZ PCR Screen	Graham et al 2021
zebra2	TGACAGCTGACGAGGATTTCT	ΔZ PCR Screen	Graham et al 2021

Development • Supplementary information

Table S8. Sequences of transgenes and constructs used.

CRE Name	Sequence
ftz_(-7) (ftz3+6) transgene	ATGTTTCTCGTTCGAGGTTGGAGTTGAATTCGGAGTCGGAGTTGGCTGGCTGGCTGCCTTGCTGGTGAAGGGATTGGCCTTCGTTTCACCCATCACCGCCAGAAAATTTCCCGGGCTGATTAGCATGAGGCGTAACAGTTTTTAATTGCAGCCACATTTCGCCGAGCTCCGAATGTGCAGCAACAAAGCCTGGCAAACACATAATGATAGCAATACTTATCTTGCCATCAGAA AGTtGAAAAGTTTAAGCACTTTTTCAATTTTTTTGTAACAACCACCAATGCTTCTATGCGACAGCTTTCCCTCTACTGTGACTTCTTTAGATGTTTTCaGCTGGATTGATATATTTTATTTCGTTTAAAAGATTGTTAAAAGAAAACacAACTAatAatGtTTTTCATACGATACATTCCATTTTATTGTAATTTTGTGTACCCATTGACAATTTTACAAAGAATACACTCCT ACAATATCACTGAACAGTTGGATAATAAAAAAACTCAGAATGTTTTTTTtAAATGAGTGTTCATAATAAACTTGAACGCAGTCTTGCCGAGTTTTTTAAGCGACAATATGTCTATTAAACATATTGATGTAGTGgCATTTAAAAATTATTTGTGCTATTAtTTTTTAAATAAGTACTCCTTTTTAGCTTTCATAGTTCCCGAGATCACATCGTTCATATGGACAGACAGATT GACAGATAAGCCCAGATTGAATTGGCCAGTAATCCCGATAAAGAATACGTATTCACTTTCATCCTTTGATACATACTTTTCATACTTTATACCCTTTTACTCTACGAGGAACGGGAGTAATTGTTGGAAATGTTTATTCACCACTCCCAGTTATTGCATTTATACAAATTATTACTTGACTAGATTTTATAATCAAAATATTTAATTAAAATCCtCCGGTCATTATGTCCTGCT CTACATATAATTTAGACCTCCCATCGGGTgTTGACTGTCCGTAGCTGTACTATCATTATCCTCCTAAGGCTGTCAAGTTATCTACGCCGCTTGAGGCCATTGTCAgTGTCTTCGCCGGAACTGCTACAAAAAATTGTGACAATTTACACTGATCTCTGGAACCTGGTCCACAGACCCGTCCTTGACTGCCATAAAGTCAGTTCCACTAAAAATCGTCGAAAAATACTCCCGTGA ACTAGAAAAAATAAAGCACACAAAAAATGTGCCTCAGTTCTAGTTTAGTTTCAAGAAATTCTCTGTTCGCTCGGAAGGAAAAAATGGAAACTAGAATACGCGGGACAATAAAAAAACGATGCGCTGTAAAAACGTTTTAGACCGAATTCACCTTTGTGTTTCGCGCATTCTCGTCATTGTTAAAAAAAATCAAGCATTTTTTTGGGTCGCGACACAATTTTTTGCCGTAGTGTC TCCAGGATTCCTCTAGATCCTGGTCTCGTTCTCAGGCCGTGGCCTCGTTTTCCATTTAAAATGCGCGACGCACAAAAGTTTTTACACGAATTTATGTTTTTATCGCTTTACGACCGGAGGTCCTAATAAAACTTTATTGAGGCTGCCGG
ftz_(-6) (ftz2+7) transgene	TGCCGGTGGTCGTAGGAGTTTTGTCCGAGTTTCTTACCGTAGTAGGCCTCCTTTGGTAGCCTCAAAACTCAAAACTGTAGCAgGGTCAGGTTCCTCGGGATAAGCGCGTATCTGCGAATTAAGCCAGCCCAAGGACGAGCCGGGTTATGCAAGTCTATGCAACGTATGGGTTTGCATAATAGAGGTAAATGTTGTTTAACTGGGAACTGCTCTAGTTAGCATTGTCTTGATG GCATTGTTTCTGCCCTCACCCGGTACCGCCCACGAGCCGGGAAGACAGTCAATAAAACGTCACCTTCACCTCTTACACAATATTTCACAACGTTCTTGCAAATCCGGGGATTAGGAACAATTCCGGCGTTCTCAAAAGGTTTTGGGCATCATGCCCTTCCGTAGTCGCCGTTTGCTCTAATTTAAATTACCATTCTGCATTGTTTTGGAATGTTTCCTAAAAACTGACCTAAT TTACGCCACACACTTTTCGTTACAAGTCTTTCGTTTTTATGACCCAAAAATGGTAAAAGTTTCCGCGAATTTAATCATTTTTATGATGTGAACGATTTTTTGAATAGATGTTGCGAATTCCGAAATCGGGAAATAGAAATATGATTAAAATAGCATTAGaCAATGCTTTGGTTATTTTTATGACTTAAATTGAGTTAGAATGTAATTTTAGATTTTAATTGAAAAATATTTAT TTAACATATAGTAACTAAGAATTTTTTTATTAATAATTATATATGTATATATATATATTATATAAGTATATTATTATATATATATTTTATTTTATTATTATATTATATATATATAATTTTTTTAGCTTTCTTATAATTTTAATATTTTTATTATATATTTTTTTTTGCAAAAGATAAATTAACAAACCAATATTTCCGCTTCACAAGCAATCCAAGCCCCGATAATAAAATG TTCTATATTTCTTcCTAAAAATATATACTATATTAATTATGTTTTATTACTATATATGTATTTATATTTGCAATGATTATTGTTCAATCGAAGAAAAAAATATTGCATGCAATACTTTGGGGTGTTCTTAGTTTTTCTGAATATATTTTTTATTTATTTTACTTTAGTTTGTTATCTTAACAAATAATCTTAATTTATAATATAGTTATAAACAAATGTTCAATCGTTTTTC CAGACAAAATGTCTTTTACATTCGCAGTTCAACATCTTAATCCTTTTGCGAAACATTACTGCCATTACAT
ftz413 (ftz2) transgene	GGTACCGCCCACGAGCCGGGAAGACAGTCAATAAAACGTCACCTTCACCTCTTACACAATATTTCACAACGTTCTTGCAAATCCGGGGATTAGGAACAATTCCGGCGTTCTCAAAAGGTTTTGGGCATCATGCCCTTCCGTAGTCGCCGTTTGCTCTAATTTAAATTACCATTCTGCATTGTTTTGGAATGTTTCCTAAAAACTGACCTAATTTACGCCACACACTTTTCGTTA CAAGTCTTTCGTTTTTATGACCCAAAAATGGTAAAAGTTTCCGCGAATTTAATCATTTTTATGATGTGAACGATTTTTTGAATAGATGTTGCGAATTCCGAAATCGGGAAATAGAAATATGATTAAAATAGCATTAGaCAATGCTTTGGTTATTTTTATGACTTAAATTGAGTTAGAAT
ftz 3' element (ftz5) transgene	CTATTTATTTAAATATACGAGTAAAGTAAATCGATCGAATTTAAACAAATCAAGTTGAACATTCATTTGGCAATTTGTGAAGAAGAGTCTTGGGCATGCTGCAATTTGACTGCTTTAAAATTTTAAATTTATAGGCCGTGGGCCGCGTATGTGGAATACATTTCATATGTATATGTATATGTTGAAATAcAATTAAATGCCTTTCAATGATAACTACTCAATAAATTCTTAAAATAGT TTGGATCAACGTATGGTATTTGTCTAACTACTTTCACCGAAAACATATCACAAGGCAAAGTTTACCTTCAGCCTATTTaCATACCTTATATATGCATATAATACATATATATGATAAAAATGGAGTTTAGGAGTTAAGGAGTTTAcATTATTTAAGTATAAGCCTAATATTTAATTGTTCGTTGAGAAGCTTAAGAGATCTCTTGGTTTACTTTCTTGATGAAgTGCACATGGTT GAAGCATTACTTTCGAACTTATACGAAACGCAAACGATTTAATGTTGAGCACGAATCGTACAAATTCGAGCAGCTGCATTTTGCTCGCTCAGCCCCCTCATCCCTGACCCATTGCTgTCTCCCGGATTTTCTATTAAATGCACTCTTTTCGCCAGAGAAAATGTCACATTTTGGTCTGGCTTCGGGGCATATCTACCACCGCATCCCTGCTCCCTTCCTCCCTCCGaCGCTGC ACGTTCCTCTATTGAAGTGAGACATTGATTGGTAATTTTTCATTGCACATCCGTGACAGTTATGGGTAACGCAACGCAAAAGGAAAAGCCCGGTGCGGAATCGGATTCGGAATCAGAATCAATATCAAAGGCAAAGGGGATCCATGTGCAGTCGAATATATCCCGGCACCATTTATCCGACTCATTAGACAAAAGTGGTCGGTGGGTGAGTTCGAAGTGGCATCTGTGGGCACT TTAGTGTTAGGTGCGAATTGGTGGCATTGGTTAAGCTTAGTGGTGATATGTATCTATGCCGCTGCGAAAGAAATTTTTTGTCTGATTTAATGATTTAGATGGCTTCAATGTGCTACTTAATTGATTTGGAAACCATGTACATTTTGTTCAAATATTTTATAAAAATAAATATTTAACAATGAACAaCCTTCAATAGATCAGATTCTATCCTACtCTTCTAGAATaTAGTTATGA CAGACAAATTAGTTAACAGATATAAATCAAAAGACCGAAAACGGAAGAATCCCTTTGCAAATTCGAATTTGTaGCATTTTTGCAATTATATAATTAGGCCATAAATATATAAAtGATGGTGGTTGGGTAAAAAAGCCCTACAGTGACATACATATCTACAATACAAACTACTTGGCAAATACAGTATGCGTCAGAAATATAAGTCAACGAAGTATTTATATTTTCTAAATAGTC AACTTTTATTAAACCCGTTACGCGTATTAGAAAAGGGTATACTTGATCCGTTGAAAAGTATGTAACAGCTAAAAGTTTTTAAAATAAAAGTTTTTTTCCGTATAAGCTAGGATTCCAGAGATGCACACACAACAATTAATACGTTGAAATTTCTTGTTCGTAATCCTACTAGGTGATAGTCGAAAAACTCGACAATACCATTCCTCTAGTTGTTTTGCAAACATCAACCAAGC GGATTTTACTGGTTCCTTTGACTCCTTAAAGTTATCAATTAGTCAACATTAAACAAACGAAATTGCAAGGACATTTACCCGTTTCTcAAAAGTCAACATCTCTTTTCATACGTTTCCTTGGAAGTTAAAGAAAAACTCAAGAAACTGGTAAAATATTTACGTGTAAATTGCGAAAGAGTTACGTTAACTCTTCCTGTGTCCTTCTGCGAAACGAAGTCTTTTTATCCTTTTA TTCGCATTCTGCTTTATTAGAGGTCCTCGGAGTCGGTTCTTAACTCATTCGCGAGCCATAAACCCCGAGATTCGCGGAAAGGGTATTCGTTGTGGGCGGAATCCTTTTGCAAACCCGGTTTCAGTCAAGGCTGCAAAGTCAATCCTTTAGCATAATCCTTTAAATAGCCAAAATAAAGCCCGCCTTTGTCAAAACAAAGCGATTCGCTGGCAGAACTCGTATTTGCGAATAGGC GTGCCATTGTGTCCACTTAGCCGGCAG
ftz_(+3) (ftz1+5) transgene	CCGCTGCACGTTCCTCTATTGAAGTGAGACATTGATTGGTAATTTTTCATTGCACATCCGTGACAGTTATGGGTAACGCAACGCAAAAGGAAAAGCCCGGTGCGGAATCGGATTCGGAATCAGAATCAATATCAAAGGCAAAGGGGATCCATGTGCAGTCGAATATATCCCGGCACCATTTATCCGACTCATTAGACAAAAGTGGTCGGTGGGTGAGTTCGAAGTGGCATCTGT GGGCACTTTAGTGTTAGGTGCGAATTGGTGGCATTGGTTAAGCTTAGTGGTGATATGTATCTATGCCGCTGCGAAAGAAATTTTTTGTCTGATTTAATGATTTAGATGGCTTCAATGTGCTACTTAATTGATTTGGAAACCATGTACATTTTGTTCAAATATTTTATAAAAATAAATATTTAACAATGAACAaCCTTCAATAGATCAGATTCTATCCTACtCTTCTAGAATaTA GTTATGACAGACAAATTAGTTAACAGATATAAATCAAAAGACCGAAAACGGAAGAATCCCTTTGCAAATTCGAATTTGTaGCATTTTTGCAATTATATAATTAGGCCATAAATATATAAAtGATGGTGGTTGGGTAAAAAAGCCCTACAGTGACATACATATCTACAATACAAACTACTTGGCAAATACAGTATGCGTCAGAAATATAAGTCAACGAAGTATTTATATTTTCTA AATAGTCAACTTTTATTAAACCCGTTACGCGTATTAGAAAAGGGTATACTTGATCCGTTGAAAAGTATGTAACAGCTAAAAGTTTTTAAAATAAAAGTTTTTTTCCGTATAAGCTAGGATTCCAGAGATGCACACACAACAATTAATACGTTGAAATTTCTTGTTCGTAATCCTACTAGGTGATAGTCGAAAAACTCGACAATACCATTCCTCTAGTTGTTTTGCAAACATCA ACCAAGCGGATTTTACTGGTTCCTTTGACTCCTTAAAGTTATCAATTAGTCAACATTAAACAAACGAAATTGCAAGGACATTTACCCGTTTCTcAAAAGTCAACATCTCTTTTCATACGTTTCCTTGGAAGTTAAAGAAAAACTCAAGAAACTGGTAAAATATTTACGTGTAAATTGCGAAAGAGTTACGTTAACTCTTCCTGTGTCCTTCTGCGAAACGAAGTCTTTTTATC CTTTTATTCGCATTCTGCTTTATTAGAGGTCCTCGGAGTCGGTTCTTAACTCATTCGCGAGCCATAAACCCCGAGATTCGCGGAAAGGGTATTCGTTGTGGGCGGAATCCTTTTGCAAACCCGGTTTCAGTCAAGGCTGCAAAGTCAATCCTTTAGCATAATCCTTTAAATAGCCAAAATAAAGCCCGCCTTTGTCAAAACAAAGCGATTCGCTGGCAGAACTCGTATTTGCG AATAGGCGTGCCATTGTGTCCACTTAGCCGGCAGGTACCGCCTCCTCAAAACCCTCGGTCGCGGAGGACTCCTGGTCTGCATCTCTATCCCTTCCACCAGCCTCAGCCATAACGATCGTAAAGTTATCCTTTCGCAAACACGAGCATtCGGTTACCGCCTTAAGCTGGCGAAGGAGTTACAAAGTCATAAAAACAAAACACCTTTGTCCGgGCAGCATTGTCAGTTGGCGAAT TATCCGTTCAGTCGCCGCTGCCGAATCCTTAATCCGTGCCGTTTTTATAGCCAGTAAGTAGATGGAGATAGTAGGAAATTCGCGATGGGCTCGTCCCTGGCGAAAGGGGAAGGAACACGACAGGC
pCFD5-ftz2	CTGTCTTCCCGGCTCGTGGGGTTTTAGAGCTAGAAATAGCAAGTTAAAATAAGGCTAGTCCGTTATCAACTTGAAAAAGTGGCACCGAGTCGGTGCTAACAAAGCACCAGTGGTCTAGTGGTAGAATAGTACCCTGCCACGGTACAGACCCGGGTTCGATTCCCGGCTGGTGCAATAGCATTAGACAATGCTTGTTTTAGAGCTAGAAATAGCAAGTTAAAATAAGGCTAGTCC GTTATCAACTTGAAAAAGTGGCACCGAGTCGGTGCTAACAAAGCACCAGTGGTCTAGTGGTAGAATAGTACCCTGCCACGGTACAGACCCGGGTTCGATTCCCGGCTGGTGCGCCACAATTGTCGATCGTCAGTTTTAGAGCTAGAAATAGCAAGTTAAAATAAGGCTAGTCCGTTATCAACTTGAAAAAGTGGCACCGAGTCGGTGC
ftz2 HDR template	GTGTACCCATTGACAATTTTACAAAGAATACACTCCTACAATATCACTGAACAGTTGGATAATAAAAAAACTCAGAATGTTTTTTTtAAATGAGTGTTCATAATAAACTTGAACGCAGTCTTGCCGAGTTTTTTAAGCGACAATATGTCTATTAAACATATTGATGTAGTGgCATTTAAAAATTATTTGTGCTATTAtTTTTTAAATAAGTACTCCTTTTTAGCTTTCATAG TTCCCGAGATCACATCGTTCATATGGACAGACAGATTGACAGATAAGCCCAGATTGAATTGGCCAGTAATCCCGATAAAGAATACGTATTCACTTTCATCCTTTGATACATACTTTTCATACTTTATACCCTTTTACTCTACGAGGAACGGGAGTAATTGTTGGAAATGTTTATTCACCACTCCCAGTTATTGCATTTATACAAATTATTACTTGACTAGATTTTATAATCAAA ATATTTAATTAAAATCCtCCGGTCATTATGTCCTGCTCTACATATAATTTAGACCTCCCATCGGGTgTTGACTGTCCGTAGCTGTACTATCATTATCCTCCTAAGGCTGTCAAGTTATCTACGCCGCTTGAGGCCATTGTCAgTGTCTTCGCCGGAACTGCTACAAAAAATTGTGACAATTTACACTGATCTCTGGAACCTGGTCCACAGACCCGTCCTTGACTGCCATAAAGT CAGTTCCACTAAAAATCGTCGAAAAATACTCCCGTGAACTAGAAAAAATAAAGCACACAAAAAATGTGCCTCAGTTCTAGTTTAGTTTCAAGAAATTCTCTGTTCGCTCGGAAGGAAAAAATGGAAACTAGAATACGCGGGACAATAAAAAAACGATGCGCTGTAAAAACGTTTTAGACCGAATTCACCTTTGTGTTTCGCGCATTCTCGTCATTGTTAAAAAAAtcaagcat TTTTTGGGTCGCGACACAATTTTTTGCCGTAGTGTCTCCAGGATTCCTCTAGATCCTGGTCTCGTTCTCAGGCCGTGGCCTCGTTTTCCATTTAAAATGCGCGACGCACAAAAGTTTTTACACGAATTTATGTTTTTATCGCTTTACGACCGGAGGTCCTAATAAAACTTTATTGAGGCTGCCGGTGGTCGTAGGAGTTTTGCTCGAGTTTCTTACCGTAGTAGGCCTCCTTT GGTAGCCTCAAAACTCAAAACTGTAGCAgGGTCAGGTTCCTCGGGATAAGCGCGTATCTGCGAATTAAGCCAGCCCAAGGACGAGCCGGGTTATGCAAGTCTATGCAACGTATGGGTTTGCATAATAGAGGTAAATGTTGTTTAACTGGGAACTGCTCTAGTTAGCATTGTCTTGATGGCATTGTTTCTGCCCTCACCCGGTAATTTTAGATTTTAATTGAAAAATATTTTA TTTAACATATAGTAACTAAGAATTTTTTTATTAATAATTATATATGTATATATATATATTATATAAGTATATTATTATATATATATTTTATTTTATTATTATATTATATATATATAATTTTTTTAGCTTTCTTATAATTTTAATATTTTTATTATATATTTTTTTTTGCAAAAGATAAATTAACAAACCAATATTTCCGCTTCACAAGCAATCCAAGCCCCGATAATAAAAT GTTCTATATTTCTTcCTAAAAATATATACTATATTAATTATGTTTTATTACTATATATGTATTTATATTTGCAATGATTATTGTTCAATCGAAGAAAAAAATATTGCATGCAATACTTTGGGGTGTTCTTAGTTTTTCTGAATATATTTTTTATTTATTTTACTTTAGTTTGTTATCTTAACAAATAATCTTAATTTATAATATAGTTATAAACAAATGTTCAATCGTTTTT CCAGACAAAATGTCTTTTACATTCGCAGTTCAACATCTTAATCCTTTTGCGAAACATTACTGCCATTACATACAGTTCAATTTCTTGTGAGACGGGGGCGTTGGCTAAAGGAAAGTCTGTTTTGGGTAAACAGAGCGAAAATCCCTTTCGCCGCCATTTCCATCCATTTCCACCTCTGATTTCTCTGATTTCACGGACGAATCAAGGCTACCTGGGAAGGGAAGAGCGCGTGTGTCT CGCATTAGACTGATGTTCTGTTGAACGGGACGGGAAATGGAAATGCGTGGCCGAATCGAGGACGAGGCTCGCGCTACTCGACTTGGACCGACTGAGTCGTCTGTTTATGGATAGTAATGCACCGTGTCATGGGTCCTTAAGCGTCTTAATTGCCCGCGTATCCTTCGCCGAGAAGCAGCGCAATTGGTTCCAAGGCGGAAATATTATTGGCACATCATAAACTATAAAAC AGGCCGCAGGGGGCCCCCTTCCTCCTTCCTTCGTTTTCCTGTCCAGTTCAGTTGAGCTCTTCACCTCGGATTGCGAGCTCTGAGATTCAAACTGAGACATCCAATGCCATCAGCGAATTCGCCGAATTTTCGAGCAGCGCGCGCGACTTCGATTCCCCGGCGTACGACTCGTCAGCGGAATCGCATTGACAGCCTACGCCGAACTCCCCGGAGACCTCCGTTCCGATCATTGT TAATGGACGTTATCCTTATTAGATGTTGATGTCCCACGATGTCGAATAGCTACGGGACATACATATCTACATATATAAAAGATATGCCCATGTTAGGTATTAAGATCTGATTCAGTTCATGTTCAGCTTCATTATATTATAGATTAAAATTATGTGAATGCTTTTATTTCTATTTCCGTTTCATCAGTTTGCATTTTATATTCAAATTGTATAATCTCTTTAAATTATTAAAAT TGTTATAGTGCACGGACTTCGAGCCTGCTTAAAGAATCGAAACTTTAAATTACTATACAAGGTACAAGGTATTAGGAGAATTAACATAGTTTCACATGACATTATTTATCAATAGTTTTTAACTGATGACACTCTGACATTTTTCAAAAAGCCATCAATCTTTGAGCTCAGTTGGAGCGTCCGTAACAGAAGGTGTTAACGAGGTCCTAGCTGAGATTTAAACAATTCGAAT TTATATCTAGGGGCCATAAGCAGTACGATGCGATGATGCACACAGTTCACATTTCATTCAGCTTACGGGGTCATCATTGCtattTAAAAGCGGCAGGACAATTCGAATAAGTGTGACAGGAGCAATTACAGCCTTATCCTGATGTCTTCGATGTCAACACACCAGTGCTCAAGACATCGCAGGCAATTCAAGGATATGTAGGACGCACAGGACCTCAACAGAAGCCAAGGACA CAGGCGACGCGTGACGCATTGGGAAAATATTTGTACAAAACATTGAGGATATCTCAAAAGTAGCACAGCGTTTCGGCATCCTTGACTTTGATTGGTGCCGATCCAAGGACGAAACGCTATATCCACCCATTGAGATGACCTCCGGAGGAGTCGTCGGTGGGCCTCCGATCGAAGGTCACCCAGAAATGCATCCTGTCTGGCTTTCTCTTCAaTtTATAGTGAATTTTAGAAACT GAAGATTACTTCATTTATCTAGA
pCFD5-ftz2+7	AGCAAAACTCCTACGACCACGTTTTAGAGCTAGAAATAGCAAGTTAAAATAAGGCTAGTCCGTTATCAACTTGAAAAAGTGGCACCGAGTCGGTGCTAACAAAGCACCAGTGGTCTAGTGGTAGAATAGTACCCTGCCACGGTACAGACCCGGGTTCGATTCCCGGCTGGTGCTGGCAGTAATGTTTCGCAAAGTTTTAGAGCTAGAAATAGCAAGTTAAAATAAGGCTAGTCC GTTATCAACTTGAAAAAGTGGCACCGAGTCGGTGCTAACAAAGCACCAGTGGTCTAGTGGTAGAATAGTACCCTGCCACGGTACAGACCCGGGTTCGATTCCCGGCTGGTGCGCCACAATTGTCGATCGTCAGTTTTAGAGCTAGAAATAGCAAGTTAAAATAAGGCTAGTCCGTTATCAACTTGAAAAAGTGGCACCGAGTCGGTGC
ftz2Δ2+7 HDR-template	GTGTACCCATTGACAATTTTACAAAGAATACACTCCTACAATATCACTGAACAGTTGGATAATAAAAAAACTCAGAATGTTTTTTTtAAATGAGTGTTCATAATAAACTTGAACGCAGTCTTGCCGAGTTTTTTAAGCGACAATATGTCTATTAAACATATTGATGTAGTGgCATTTAAAAATTATTTGTGCTATTAtTTTTTAAATAAGTACTCCTTTTTAGCTTTCATAG TTCCCGAGATCACATCGTTCATATGGACAGACAGATTGACAGATAAGCCCAGATTGAATTGGCCAGTAATCCCGATAAAGAATACGTATTCACTTTCATCCTTTGATACATACTTTTCATACTTTATACCCTTTTACTCTACGAGGAACGGGAGTAATTGTTGGAAATGTTTATTCACCACTCCCAGTTATTGCATTTATACAAATTATTACTTGACTAGATTTTATAATCAAA ATATTTAATTAAAATCCtCCGGTCATTATGTCCTGCTCTACATATAATTTAGACCTCCCATCGGGTgTTGACTGTCCGTAGCTGTACTATCATTATCCTCCTAAGGCTGTCAAGTTATCTACGCCGCTTGAGGCCATTGTCAgTGTCTTCGCCGGAACTGCTACAAAAAATTGTGACAATTTACACTGATCTCTGGAACCTGGTCCACAGACCCGTCCTTGACTGCCATAAAGT CAGTTCCACTAAAAATCGTCGAAAAATACTCCCGTGAACTAGAAAAAATAAAGCACACAAAAAATGTGCCTCAGTTCTAGTTTAGTTTCAAGAAATTCTCTGTTCGCTCGGAAGGAAAAAATGGAAACTAGAATACGCGGGACAATAAAAAAACGATGCGCTGTAAAAACGTTTTAGACCGAATTCACCTTTGTGTTTCGCGCATTCTCGTCATTGTTAAAAAAAtcaagcat TTTTTGGGTCGCGACACAATTTTTTGCCGTAGTGTCTCCAGGATTCCTCTAGATCCTGGTCTCGTTCTCAGGCCGTGGCCTCGTTTTCCATTTAAAATGCGCGACGCACAAAAGTTTTTACACGAATTTATGTTTTTATCGCTTTACGACCGGAGGTCCTAATAAAACTTTATTGAGGCGGTACCGCCCACGAGCCGGGAAGACAGTCAATAAAACGTCACCTTCACCTCTTA CACAATATTTCACAACGTTCTTGCAAATCCGGGGATTAGGAACAATTCCGGCGTTCTCAAAAGGTTTTGGGCATCATGCCCTTCCGTAGTCGCCGTTTGCTCTAATTTAAATTACCATTCTGCATTGTTTTGGAATGTTTCCTAAAAACTGACCTAATTTACGCCACACACTTTTCGTTACAAGTCTTTCGTTTTTATGACCCAAAAATGGTAAAAGTTTCCGCGAATTTAATC ATTTTTATGATGTGAACGATTTTTTGAATAGATGTTGCGAATTCCGAAATCGGGAAATAGAAATATGATTAAAATAGCATTAGaCAATGCTTTGGTTATTTTTATGACTTAAATTGAGTTAGAATGTAATTTTAGATTTTAATTGAAAAATATTTTGGGTAAACAGAGCGAAAATCCCTTTCGCCGCCATTTCCATCCATTTCCACCTCTGATTTCTCTGATTTCACCTCTGATTTCT CTGATTTCACGGACGAATCAAGGCTACCTGGGAAGGGAAGAGCGCGTGTGTCTCGCATTAGACTGATGTTCTGTTGAACGGGACGGGAAATGGAAATGCGTGGCCGAATCGAGGACGAGGCTCGCGCTACTCGACTTGGACCGACTGAGTCGTCTGTTTATGGATAGTAATGCACCGTGTCATGGGTCCTTAAGCGTCTTAATTGCCCGCGTATCCTTCGCCGAGAAGCAG CGCAATTGGTTCCAAGGCGGAAATATTATTGGCACATCATAAACTATAAAACAGGCCGCAGGGGGCCCCCTTCCTCCTTCCTTCGTTTTCCTGTCCAGTTCAGTTGAGCTCTTCACCTCGGATTGCGAGCTCTGAGATTCAAACTGAGACATCCAATGCCATCAGCGAATTCGCCGAATTTTCGAGCAGCGCGCGCGACTTCGATTCCCCGGCGTACGACTCGTCAGCGGAATC ATCGCATTGACAGCCTACGCCGAACTCCCCGGAGACCTCCGTTCCGATCATTGTTAATGGACGTTATCCTTATTAGATGTTGATGTCCCACGATGTCGAATAGCTACGGGACATACATATCTACATATATAAAAGATATGCCCATGTTAGGTATTAAGATCTGATTCAGTTCATGTTCAGCTTCATTATATTATAGATTAAAATTATGTGAATGCTTTTATTTCTATTTCCGTT TCATCAGTTTGCATTTTATATTCAAATTGTATAATCTCTTTAAATTATTAAAATTGTTATAGTGCACGGACTTCGAGCCTGCTTAAAGAATCGAAACTTTAAATTACTATACAAGGTACAAGGTATTAGGAGAATTAACATAGTTTCACATGACATTATTTATCAATAGTTTTTAACTGATGACACTCTGACATTTTTCAAAAAGCCATCAATCTTTGAGCTCAGTTGGAGCG TCCGTAACAGAAGGTGTTAACGAGGTCCTAGCTGAGATTTAAACAATTCGAATTTATATCTAGGGGCCATAAGCAGTACGATGCGATGATGCACACAGTTCACATTTCATTCAGCTTACGGGGTCATCATTGCtattTAAAAGCGGCAGGACAATTCGAATAAGTGTGACAGGAGCAATTACAGCCTTATCCTGATGTCTTCGATGTCAACACACCAGTGCTCAAGACATCGC AGGCAATTCAAGGATATGTAGGACGCACAGGACCTCAACAGAAGCCAAGGACACAGGCGACGCGTGACGCATTGGGAAAATATTTGTACAAAACATTGAGGATATCTCAAAAGTAGCACAGCGTTTCGGCATCCTTGACTTTGATTGGTGCCGATCCAAGGACGAAACGCTATATCCACCCATTGAGATGACCTCCGGAGGAGTCGTCGGTGGGCCTCCGATCGAAGGTCACC CAGAAATGCATCCTGTCTGGCTTTCTCTTCAaTtTATAGTGAATTTTAGAAACTGAAGATTACTTCATTTATCTAGA
eve stripe 2 MSE	GGTTACCCGGTACTGCATAACAATGGAACCCGAACCGTAACTGGGACAGATCGAAAAGCTGGCCTGGTTTCTCGCTGTGTGTGCCGTGTTAATCCGTTTGCCATCAGCGAGATTATTAGTCAATTGCAGTTGCAGCGTTTCGCTTTCGTCCTCGTTTCACTTTCGAGTTAGACTTTATTGCAGCATCTTGAACAATCGTCGCAGTTTGGTAACACGCTGTGCCATACTTTCATT TAGACGGAATCGAGGGACCCTGGACTATAATCGCACAACGAGACCGGGTTGCGAAGTCAGGGCATTCCGCCGATCTAGCCATCGCCATCTTCTGCGGGCGTTTGTTTGTTTGTTTGCTGGGATTAGCCAAGGGCTTGACTTGGAACCCAATCCCGATCCCTAGCCCGATCCCAATCCCAATCCCAATCCCTTGTCCTTTTCATTAGAAAGTCATAAAAACACATAATAATGATG TCGAAGGGATTAGGGG
pCFD5-eve MSE	gggctttgagtgtgtgtagacatcaagcatcggtggttcagtggtagaatgctcgcctgccacgcgggcggcccgggttcgattcccggccgatgcaATGGAACCCGAACCGTAACTgttttagagctagaaatagcaagttaaaataaggctagtccgttatcaacttgaaaaagtggcaccgagtcggtgctaacaaagcaccagtggtctagtggtagaatagtaccctgc cacggtacagacccgggttcgattcccggctgGTGCACACATAATAATGATGTCGAgttttagagctagaaatagcaagttaaaataaggctagtccgttatcaacttgaaaaagtggcaccgagtcggtgctaacaaagcaccagtggtctagtggtagaatagtaccctgccacggtacagacccgggttcgattcccggctggtgcGCCACAATTGTCGATCGTCAGtttt agagctagaaatagcaagttaaaataaggctagtccgttatcaacttgaaaaagtggcaccgagtcggtgc
eve MSE HDR template	GTTTTGGGAATCTGTCATGAATTTCTGAAATAGGTTTGCCAAACAAATTTTAAGTATAAATGTATACATATGTCAACTAATAAATTTAGCAAATAAAATGTACCTGCAAGTATCTATAAATTTATTGGACCAATTTTGTTGTAAAAAACTGAACTGGCACTCTTCCCAAGAATGGGACTTCGAGGACTCCTTGCTGAATCACTTACTCAACCCATTCCAACTCATCCAATCCG CGCAATCATCATAAATaTTGGCCTTTTTGTTGTAATTGTTTTATGGCAGAAATTACTCAATCATCAAGCATAATTCCCTCGTTTTCGCCGTTTTATTGCCAATTTTTGCACTGCCTTTGCCTTTTTCCCGCCCTTTCCTCAGCGTTTTGCGAATCTTTGCCGGCATTTCTATTGCGCGGACAATCCGGCCAGTGTGTTtGCCATTTACTTGCCATGATGACGGGCATAATCAGC GAGATCGGCGCTTTGTGAGTGCAGAATGTGCAATAAAGCGGCAACAATCGGCAGGGATTCGCCTTCCCATATTCCGGGTATTTGCCGGGCGGAAATGCGAAAGTGTTTGCGGATGGAATGGAGATAGAGGATTGAGTATTGAAAaGAGGCAGGTATTTCCTTCGCCGGCAGACACTTTCGCCTAACCAAGCCAATCCAACCCATCCCAATCCAATCCAACCCAACCCGATCGCC ATAAAGGGTATTTACTGTCGCTGCCGCAGAGCCTCGCTTGACGACTTAACCCAAGCGGTCGTTTCGCGTCCATTCTCCGGACGGAGTCAAAGACAAAGGCCGGCGGAGGTGGACAATAGGCAAGGTTGTTGCTTGTGGGTAGGGTTTGAGCTATGAGCTgTGAGCTGTGAGtTGTTAGCCCTGAACCCCGAACCTCGAGAATTGAACCTTTCCCCGGGGCAGAAGGCTTGCATG TGGGCCTTTCCAGGTCGGCCAGTAGGTAGAGTTGTTGCGATGCGGCTATGCCGGGCGAGTTAATGCCAATGCAAATTGCGGGCGCAATATAACCCAATAATTTGAAGTAACTGGCAGGAGCGAGTTACCTTCCTCGCGCAGGTCCAGGCAACGCAATTAACGGACTAGCAACTGGGTTAttttttGCGCCGACTTAGCCCTGATCCGCGAGCTTAACCCGTTTTGAGCCG GGCAGCAGGTAGTTGTGGGTGGACCCCACGACTTTTTTGGCCcAACCTCCAAGCTAACTTGCGCAAGTGGCAAGTGGCGGTTTGCTGGCCCAAAAGAGGAGGCACTATCCCGGTCCTGGTACAGTTGGTACGCTGGGAATGATTATATCATCATATAAATGTTTTGCCCAACGAAACCGAAAACTTTCAAATTAAGTCCCGGCAACTGGGTCCCATTTTCCATTTTCCAT GTTCTGCGGcCAGGGGCGGCCATTCATTCTcaCTACAGCAGTTCCCAAATGGTTATGGCTGGACACCCCTCGCCGCCGCTCCAACGGGGTGGATGAAGCCCCCAAAACCCGAAAGTCATGGCAGCCATGGCAGTGTGGGGCTGTTAAACGTGCAATAATTAAGACTTCATAAAAGCGCAAATTCGCTGGCAGGCGATCGATAATAaATACATACAAATATATAGTGGGAT ACACACACTCTCTGCCGGCAAACACACACCACCCGACCGACTGAGCGGCATAATGCCATATCATTCTTGATGAAGCCGATAAAATCCCATTATTAAGGGGGCCCGCCCGTCCCGCTCGCTCCTGCGGAGCAgCCGCCTGCGGGCGGCGAGACAAAGATTCGCTCATCCGCTATGAATACCAAATCGGAActCTCTCTCTCTCTCCAGCTCGGGAGTGCCATGGCCAGCATG GCCAGGACCTCCTCATGGTCCTGCCGAGCAGAGAACGCGGCTCCATCCCGCTGCTCCGGGTCCTGCTCCTtCGCTTTGTCCCGCCTCGTTATCGCCGCTCAGCACCGAGAGCACAGCAGCGCATCCACTCTCAGCACCGCACGATTAGCACCGTTCCGCTCAGGCTGTCCCGCTCGCACCTGCCTGGGTCGCTGCG
